# Lower Back Injury Prevention and Sensitization of Hip Hinge with Neutral Spine Using Wearable Sensors during Lifting Exercises

**DOI:** 10.3390/s21165487

**Published:** 2021-08-14

**Authors:** Florian Michaud, Manuel Pérez Soto, Urbano Lugrís, Javier Cuadrado

**Affiliations:** Laboratory of Mechanical Engineering, University of La Coruña, 15403 Ferrol, Spain; manuel.perez.soto@udc.es (M.P.S.); urbano.lugris@udc.es (U.L.); javier.cuadrado@udc.es (J.C.)

**Keywords:** injury prevention, sport performance, lifting exercises, motion capture, inertial sensor

## Abstract

The popularization and industrialization of fitness over the past decade, with the rise of big box gyms and group classes, has reduced the quality of the basic formation and assessment of practitioners, which has increased the risk of injury. For most lifting exercises, a universal recommendation is maintaining a neutral spine position. Otherwise, there is a risk of muscle injury or, even worse, of a herniated disc. Maintaining the spine in a neutral position during lifting exercises is difficult, as it requires good core stability, a good hip hinge and, above all, observation of the posture in order to keep it correct. For this reason, in this work the authors propose the prevention of lumbar injuries with two inertial measurement units. The relative rotation between two sensors was measured for 39 voluntary subjects during the performance of two lifting exercises: the American kettlebell swing and the deadlift. The accuracy of the measurements was evaluated, especially in the presence of metals and for fast movements, by comparing the obtained results with those from an optical motion capture system. Finally, in order to develop a tool for improving sport performance and preventing injury, the authors analyzed the recorded motions, seeking to identify the most relevant parameters for good and safe lifting execution.

## 1. Introduction

The popularization and industrialization of fitness over the past decade, with the rise of low-cost big box gyms and big group classes, has reduced the quality of the basic formation and assessment of practitioners, which has increased the risk of injury. After interviewing 30 sport educators from centers in France and Spain, more than 80% admitted to having clients suffering from low back pain because of their sport practice, which is common in weightlifters [1]. For most lifting exercises, a universal recommendation is maintaining a neutral spine position [2]. Otherwise, there is a risk of muscle injury or, even worse, of a herniated disc [3]. Maintaining the spine in a neutral position during lifting exercises is difficult, as it requires good core stability, a good hip hinge with lumbopelvic dissociation and, above all, observation of the posture in order to keep it correct. Although most sport centers are equipped with mirrors, some exercises do not allow the practitioner to keep his head up, as this can produce neck pain and, anyway, as in the case of posture assessment by a monitor, both are subjective.

Over the past couple of decades, various tools have been developed to improve the analysis of human motion and sport performance. Generally, these tools are used to track the motion of segments of the body so that joint angles, torques, reactions, etc., can be estimated [4]. Technological advances have improved the accuracy of these tools over time [4,5,6]. Optical motion capture systems remain the gold standard reference for biomechanical analysis because of their precision [7], but they present a lot of issues such as marker occlusions, limited capture zone, and incorrect identification of markers due to other reflective objects. All these drawbacks have led to a need for some manual post-processing of the captured data, making the technique complex [8,9]. Moreover, price and technology limit its use to researchers and specialized laboratories. Recent developments in inertial measurement units (IMUs) enable the recording of human movement in 3D [10] at a cost and complexity that are affordable to any sport center. An IMU is composed by a triaxial accelerometer, a gyroscope and a magnetometer, which allow for the estimation of its own orientation within an Earth-fixed frame by using sensor fusion algorithms, such as Madgwick’s algorithm [11] or the extended Kalman filter (EKF) [12]. Because IMUs are wearable and relatively inexpensive, over the last decade, numerous IMU-based applications for sport have emerged, either in team sports [13,14,15] or in individual sports [16,17,18,19,20,21]. IMUs have become a useful tool to evaluate the quality of movement performance and to offer quantitative feedback for sport coaching and training, which still largely rely on visual perception.

However, all these applications focus on sport performance and intend to help competitive athletes, thus forgetting the biggest part of the 184 million gym members [22], i.e., the common athletes. For this reason, in this work the authors evaluate a low-cost approach to prevent low back injuries in the gym centers by using only two IMUs. Attachments of the ribs to the transverse processes and vertebral bodies make the thoracic region the most rigid part of the spine [23]. Maintaining the spine in a neutral position mainly means avoiding lumbar motion. That is why we decided to locate the first IMU at vertebra L5 with a belt, and the second one at vertebra T8 with a harness so as to measure the relative motion between them and estimate the lumbar flexion. Authors captured the motion of 39 voluntary subjects (from several sport centers, and with different experiences, ages, and genders) during the performance of two lifting exercises of different intensity, the American kettlebell swing and the deadlift, identified by the interviewed coaches as the most likely to generate lower back pain. The accuracy of the measurements was evaluated, especially in the presence of metals and for fast movements, by comparing the obtained results with those coming from an optical motion capture system. Finally, in order to develop a tool for improving sport performance and preventing injury, the authors analyzed the recorded motions seeking to identify the most relevant parameters for good and safe lifting execution.

## 2. Materials and Methods

### 2.1. Lifting Techniques

#### 2.1.1. Hip Hinge and Lumbopelvic Dissociation

**Figure 1:**
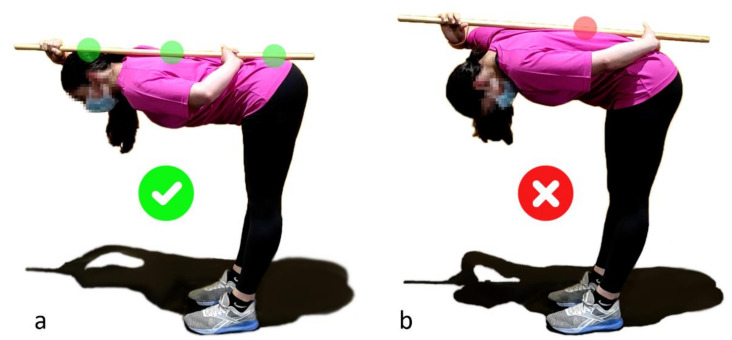
(**a**): Hip hinge with neutral spine; (**b**): Hip hinge without neutral spine.

There are numerous gym exercises (lower and upper body exercises) that require the maintenance of a rigid trunk. The hip hinge is the movement when the pelvis and upper body bend downwards driven from the hip joint. A tight core allows one to keep the spine in neutral configuration throughout the entire movement by stabilizing it from all external forces. In addition to the need for a strong core, a big problem that a lot of people face is figuring out how to flex and extend the hips without moving the lower back along with them. The lumbopelvic dissociation is a common limiting factor [24], but a major one to be overcome before lifting weights without suffering low back injuries. A common exercise to improve the hip hinge consists of using a dowel rod, as shown in Figure 1, keeping the dowel in contact with the back with the help of the hands while bending and making sure that the dowel remains in contact at the three initial contact points (back of the head, thoracic spine, and sacrum) throughout the entire movement. The dowel physically represents the pelvis-thoracic-head alignment of the neutral position and the contact points offer a feedback to the athlete, but keeping the hands at the back to hold it results in discomfort and does not allow one to lift weights. Moreover, this tool, in the same way as the subjective coach observation, does not take into account the differences between the spine shapes of the subjects [25] (especially at low back level due to acetabular version [26]) and, consequently, allows more lumbar flexion for people with more pronounced lordoses. For this reason, the authors believe that wearable sensors could offer objective feedback regarding back alignment to athletes during lifting exercises, thus helping them to improve their technique and prevent injuries.

#### 2.1.2. American Kettlebell Swing and Deadlift

While in theory books there is a unique way to do each lifting exercise, in gym centers an infinite number of methods of execution can be observed, and most of them are acceptable. The variability among subjects mainly depends on the instructions received (if received, as many people reproduce exercises seen in magazines or videos), which directly depends on the trainer and the subject’s level of experience and physical characteristics. For this reason, a wide range of subjects were chosen for this study from several sport centers with different experiences, ages, sizes and genders.

**Figure 2:**
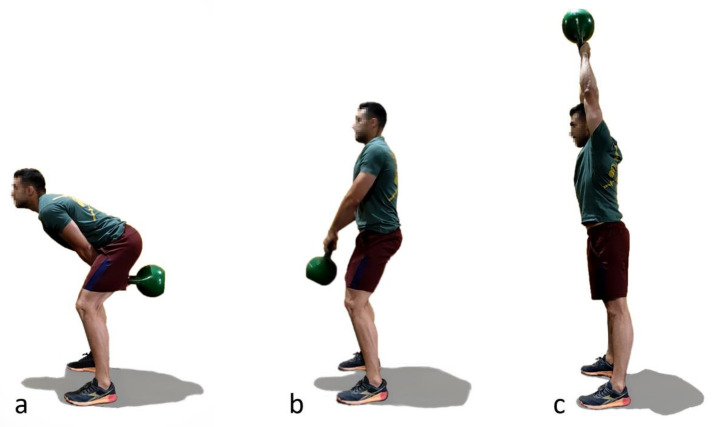
American kettlebell swing technique. (**a**): Step 1; (**b**): Step 2; (**c**) Step 3.

The first lifting exercise, called American kettlebell swing, consists of grasping a kettlebell with both hands with stretched arms and driving it from the top of the head to behind the thighs, bending the trunk rigidly (tight core) from the hip joint and squatting slightly to create an arching trajectory (Figure 2). Because it is a complete and highly intensive exercise which demands effort from muscles of the whole body, it has become very popular thanks to CrossFit. While some faults can be tolerated (too much squat, not enough bend or partial amplitudes) because they only affect muscle force distribution, an incorrect spinal position may lead to excessive disc compression and lower back injuries. Unfortunately, as pointed out by the interviewed coaches, this fault is common during the exercise due to the need for trunk bending and the high weight inertia that generates fast movements.

**Figure 3:**
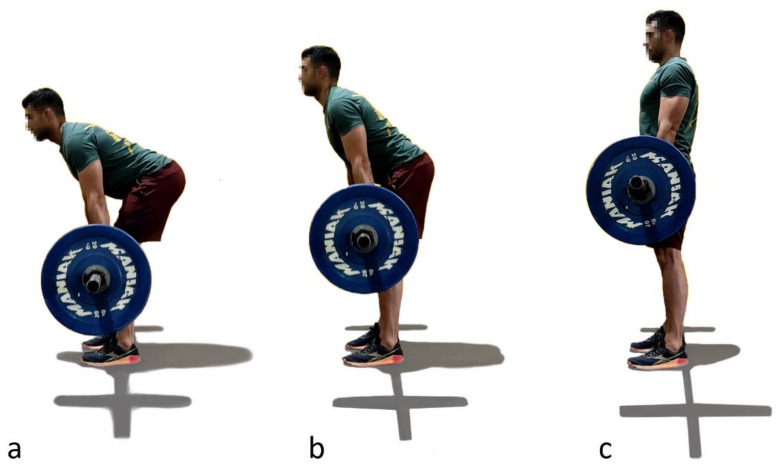
Deadlift technique. (**a**): Step 1; (**b**): Step 2; (**c**) Step 3.

The second exercise is called a deadlift, referring to the lifting of a dead (without momentum) weight, such as weights lying on the ground. It is one of the most powerful tools for an athlete to improve his/her overall strength, because it works almost every muscle in the body. As indicated by its name, it consists of lifting, vertically and slowly, a barbell from the ground, bending the trunk rigidly (tight core) and squatting slightly to the thighs through a hip hinge until putting the chest in its neutral position (Figure 3). Depending on the size of the disc, the subject’s height and the way he is doing the exercise, the athlete either touches the ground with the discs on each repetition or only lowers the bar below the knees. The faults mentioned for the previous exercise can be observed for this one too, and, because the weights lifted with the barbell are significant, an incorrect spinal position is especially dangerous when doing deadlift.

### 2.2. Preliminary Test

Because ferromagnetic materials near IMUs can disturb the local magnetic field and therefore the orientation estimation [27], it is usually recommended to avoid them when using inertial sensors. However, it is almost impossible to avoid the presence of metals in a gym because of machines, bars, dumbbells and discs. Hence, to evaluate the sensitivity of the sensors, a preliminary test was conducted to see whether specific care should be taken or a specific algorithm [27] should be implemented for this study.

#### 2.2.1. Static Perturbations

**Figure 4:**
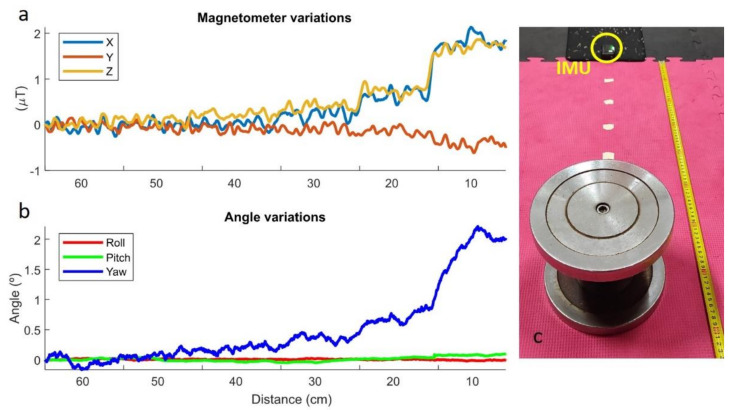
Static perturbations. (**a**): Magnetometer variations; (**b**): Angle variations; (**c**) Experimental measurements.

An IMU was positioned on the ground, and a 10-kg dumbbell was positioned at a distance of 60 cm from the sensor. Every 10 s, the dumbbell was moved 10 cm closer to the sensor, from the initial 60 cm to the final 10 cm. The orientations were computed by an in-house developed Madgwick’s algorithm [11] written in Matlab (version R2020b, MathWorks, Natick, MA, USA). Disturbances are clearly noticeable for distances lower than 30 cm, but only for distances lower than 10 cm does the error in the yaw angle exceed 1°, while pitch and roll angles showed very little error during the test, as illustrated in Figure 4.

#### 2.2.2. Dynamic Perturbations

**Figure 5:**
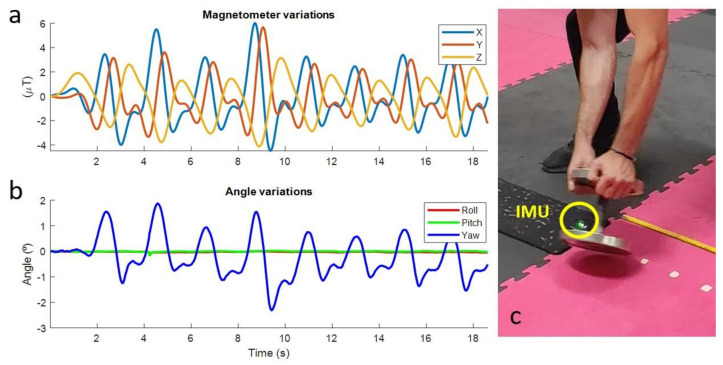
Dynamic perturbations. (**a**): Magnetometer variations; (**b**): Angle variations; (**c**) Experimental measurements.

An IMU was attached to the floor, and a 10-kg dumbbell was repeatedly moved around the sensor along the trajectory of a circle with a radius of 15 cm centered on the sensor. As can be seen in Figure 5, disturbances are clearly perceptible and generate errors in the yaw angle that range between −2° and 2°, while the pitch and roll angles (the most important ones for the application) keep their values during the test.

During the captured exercises, the metal tools were kept at a distance from the sensors of more than 30 cm at all times and, hence, no perturbations were expected. Moreover, the study focused on lower back flexion, so that only rotations in the sagittal plane were analyzed. Therefore, vertical rotation errors should not affect the results, because the couple gyroscope-accelerometer should guarantee accurate pitch and roll measurements. Finally, as the measurements from the IMUs during the lifting exercises were compared to those from the optical system (which is not affected by such disturbances), ferromagnetic disturbances would have been detected.

### 2.3. Experimental Data Collection

**Figure 6:**
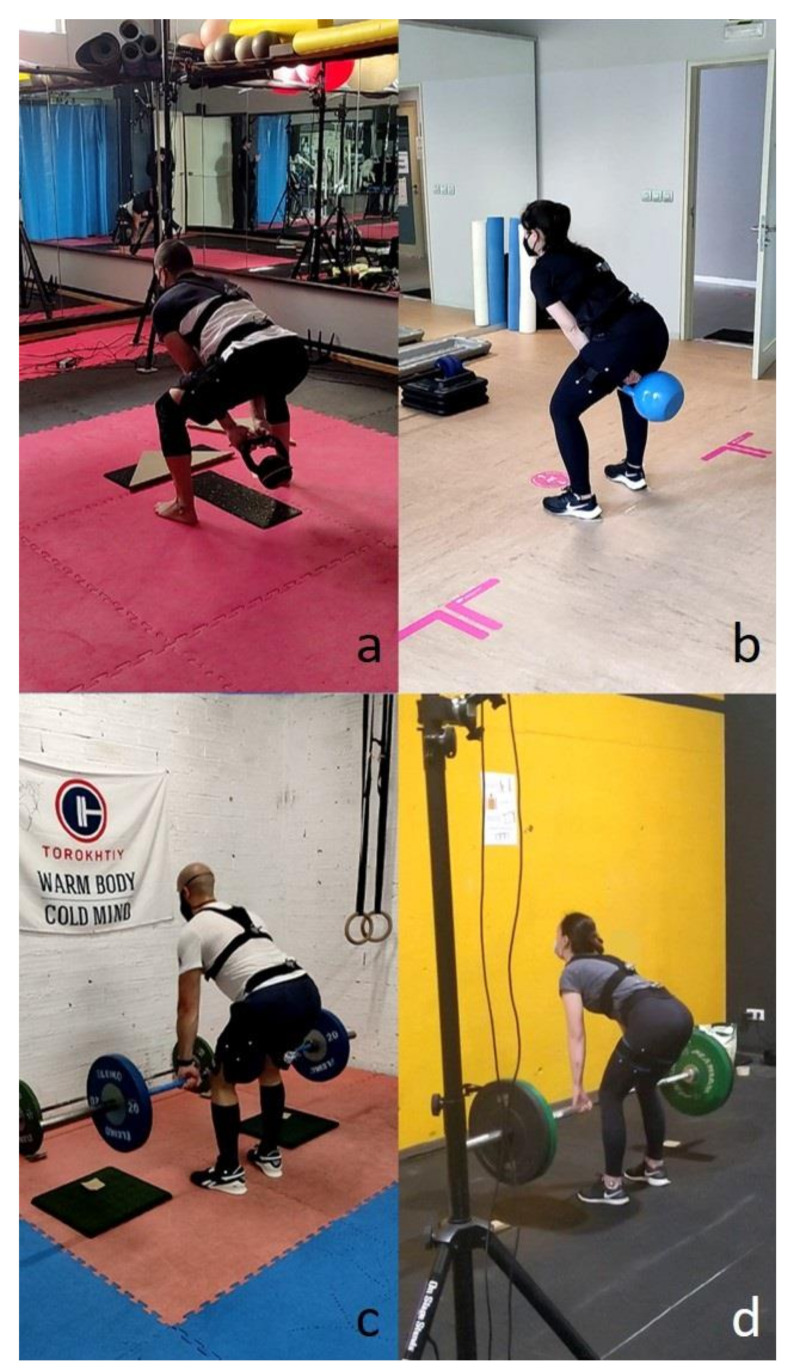
Experimental measurements during lifting exercises in four gym centers. (**a**,**b**): During American kettlebell swing; (**c**,**d**): During deadlift.

**Figure 7:**
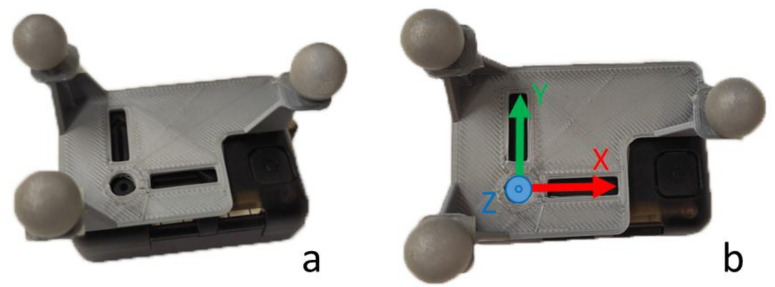
(**a**): IMU with 3D-printed plate with three markers; (**b**): IMU reference frame.

**Figure 8:**
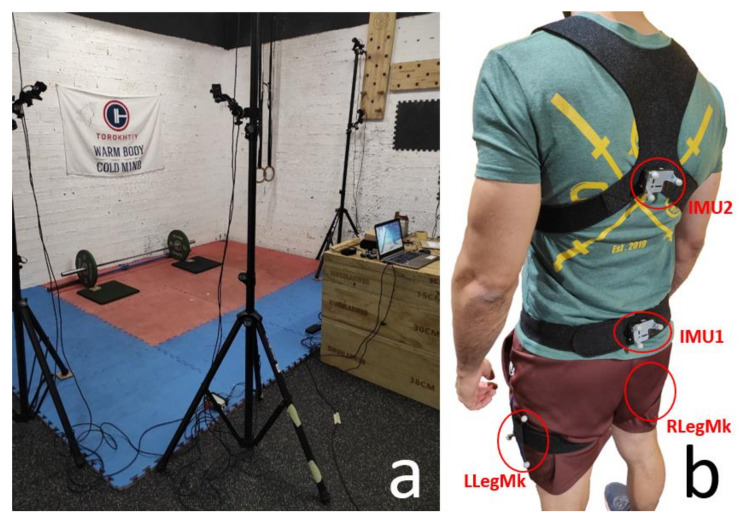
(**a**) Portable motion capture system. (**b**) Configuration of sensors.

Thirty-nine subjects (twenty-one males, eighteen females, age 39 ± 21 years, height 176 ± 22 cm, body mass 79 ± 32 kg) without injuries that could affect performance or worsen as a result of the exercise were recruited for this study. Experiments were conducted in four different sport centers with their respective members (Figure 6) of different experience levels, classified as follows: A: 0 to 6 months; B: 6 months to 2 years; C: more than 2 years. All subjects gave written informed consent for their participation. Subjects performed a static pose for 3 s (with hands free), then took the corresponding material to perform 10 repetitions of the American kettlebell swing first, and, after a resting period, deadlift (with their usual self-selected weight). Two IMUs (STT-IWS, STT Systems, San Sebastián, Spain) sampling at 100 Hz were attached to each subject’s body at vertebra L5 by means of a belt and at vertebra T8 by means of an adjustable harness (approximated locations) to measure pelvis and trunk orientations. In addition, a 3D-printed plastic plate with three markers (Figure 7) was attached to each IMU to evaluate its accuracy, and another plate with three markers was attached to each leg to measure its orientation (RLegMk and LLegMk in Figure 8b). The motion of the markers was captured using a portable optical motion system composed of eight optical infrared cameras (OptiTrack FLEX 3, also sampling at 100 Hz; Natural Point, Corvallis, OR, USA) (Figure 8a) and the trajectories of the markers were filtered with a low-pass filter (forward-backward 2nd order Butterworth filter) using a cutoff frequency of 15 Hz. Before commencing the respective data collections, the subjects completed a 10 min warm up and carried out a series of corresponding exercises to ensure that the sensors attached to their bodies did not hinder their performance.

### 2.4. Calculation of Kinematic Parameters

Orientations from IMUs were computed by a Matlab (version R2020b, MathWorks, Natick, MA, USA) implementation of Madgwick’s algorithm [11], because the commercial algorithm accompanying the sensors was shown to be inaccurate in a previous study [28]. The programmed algorithm provides an estimate of the rotation matrix by combining the information from the triaxial accelerometer, gyroscope and magnetometer present in the IMU. Orientations from the optical motion capture were computed by calculating the rotation matrix defined by the three markers attached to each body through an in-house developed algorithm also written in Matlab.

Both matrix rotations were converted to Euler angles in the rotation sequence XYZ, the rotation angles being X (normal to the sagittal plane), Y (normal to the transversal plane) and Z (normal to the frontal plane), to prevent gimbal lock [29]. Because motions were mainly two-dimensional, with frontal and transversal angle variations being irrelevant [21], the study focused on the lower back flexion and only rotations in the sagittal plane were analyzed.

**Figure 9:**
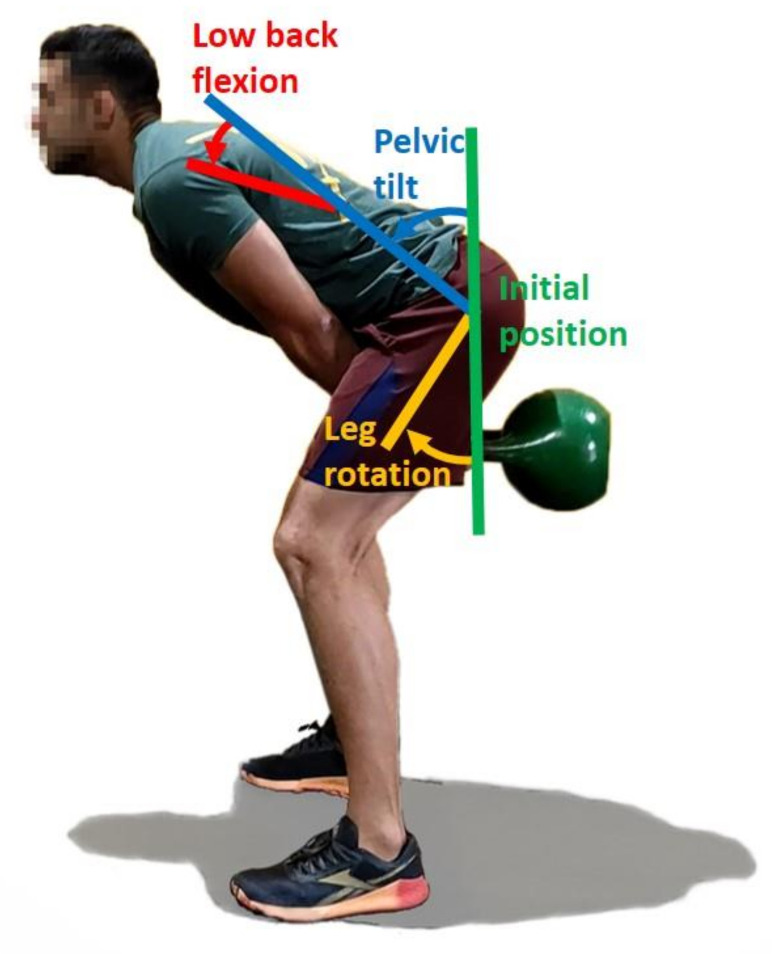
Kinematic parameters.

The initial Euler angles around X of sensor 1 (at L5) and sensor 2 (at T8), corresponding to the subject’s spine neutral position, were determined during the captured static pose and cancelled from the measured angles during motion (Figure 9, in green). The lower back flexion corresponds to the relative rotation between the chest and the pelvis (Figure 9, in red). In the same way, the angles of the right and left leg were set to 0° at the beginning of the motion. The leg rotation (Figure 9, in yellow) was used to represent the squatting of the subject instead of the knee flexion, because shank information was missing.

### 2.5. Motion Analysis and Statistical Differences

Since one objective of this study was to develop a tool that may serve to improve sport performance and prevent injuries, the authors analyzed the recorded motions seeking to identify the most relevant parameters for a good and safe lifting execution. Although the neutral zone of the whole lumbar region in healthy adults was approximately 20° in the sagittal plane [30], based on results showed in [2] the accepted neutral spine deviation was fixed to 30°. Sensors at L5 and T8 offered the required information for spine deviation and the additional markers on the legs were used to observe the squat amplitude and the possible relation with poor spine posture. Finally, in order to determine whether gender and experience level presented significant differences, several hypotheses were evaluated through a two sample t-test [31].

## 3. Results

**Figure 10:**
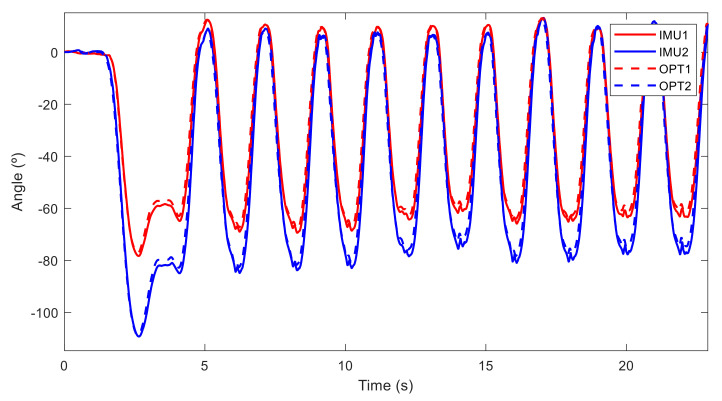
Angles measured by sensors in the sagittal plane from a woman with experience B performing deadlifts with a neutral spine position. IMU1 and IMU2 are the measurements from inertial sensor 1 (at L5) and sensor 2 (T8), respectively, and OPT1 and OPT2 are the measurements from optical motion system and markers situated at L5 and T8, respectively.

**Figure 11:**
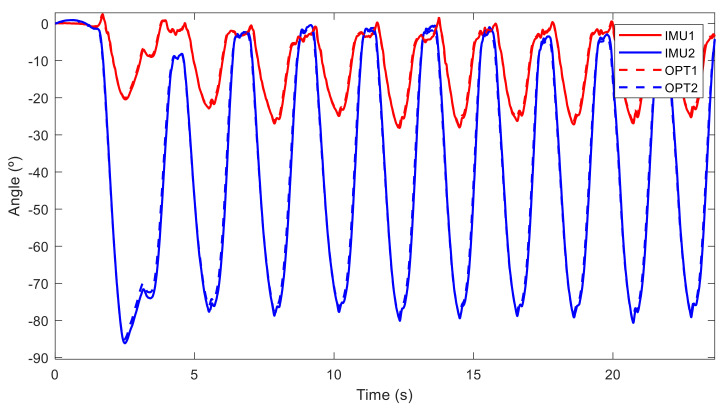
Angles measured by sensors in the sagittal plane from a man with experience C performing deadlifts without a neutral spine position. IMU1 and IMU2 are the measurements from inertial sensor 1 (at L5) and sensor 2 (T8), respectively, and OPT1 and OPT2 are the measurements from optical motion system and markers situated at L5 and T8, respectively.

The measured angles in the sagittal plane of a subject (woman, experience B) performing a deadlift with neutral spine are represented in Figure 10, while those of a subject (man, experience C) performing deadlift without neutral spine are represented in Figure 11. The first and highest pick value corresponds to the moment when the subject grasped the barbell resting on the ground, followed by the repetitions of the exercise, whereas in both cases, sensors at thoracic level (in blue) measured similar angle evolutions (reaching 80°), significant differences can be observed between the two subjects at pelvis level (in red). The first subject (Figure 10) performed a pelvic tilt between 60° and 70° at each repetition, while the second one did not exceed 30°. Measurements from inertial sensors (IMU) and the optical motion system (OPT) were almost the same.

**Figure 12:**
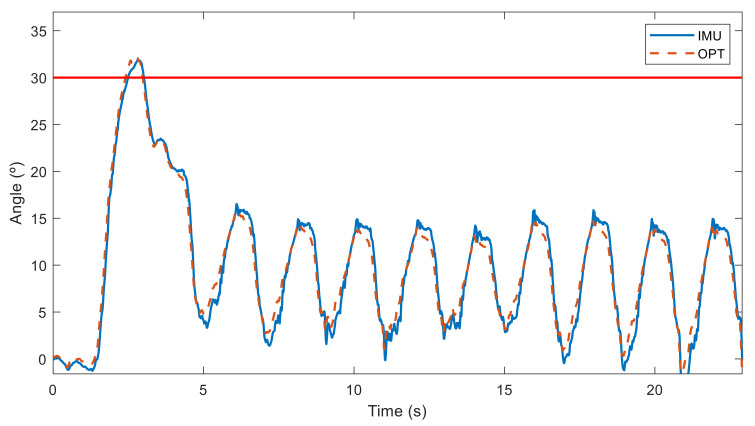
Angles measured by sensors in the sagittal plane from a woman with experience B performing deadlifts with a neutral spine position (maximum accepted spine deviation in solid red).

**Figure 13:**
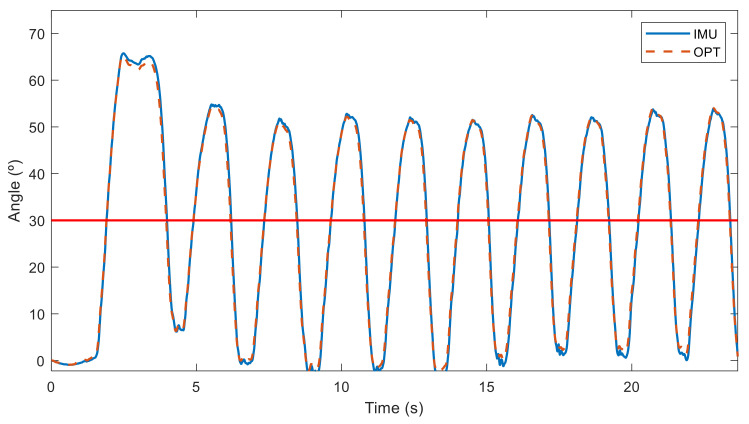
Angles measured by sensors in the sagittal plane from a man with experience C performing deadlifts with a neutral spine position (maximum accepted spine deviation in solid red).

The relative angles in the sagittal plane between the two sensors obtained with both technologies (IMU and OPT) are represented in Figure 12 for a subject doing it right (woman with experience B) and in Figure 13 for a subject doing it poorly (man with experience C). Although a deviation from the neutral spine is observed for both subjects, the first subject performed her repetitions with a low deviation (under 18°), and she only went over 30° when she bent down to grasp the barbell on the ground at the beginning. Conversely, the second subject exceeded the accepted limit during all his deadlift repetitions, with a deviation of over 50°. Measurements from the inertial sensors (IMU) and the optical motion system (OPT) were almost the same.

The maximum spine deviation and maximum pelvic tilt during the repetitions (handling of the tool is not considered) obtained through the two technologies (IMU and OPT) for all 39 subjects are represented in Table 1 for the American kettlebell swing and in Table 2 for the deadlift. During the first exercise, 11 subjects with poor execution (spine deviation exceeding the limit of 30°) were detected (in red) by both systems (IMU and OPT). Four subjects with a deviation close to the limit were only considered poor when measuring with sensors (IMU). All subjects with a high pelvic tilt (more than 65°, in blue) showed a reduced spine deviation (less than 22° with OPT). Six out of the seven subjects with a leg rotation higher than 50° (in yellow) in the squat movement were inexperienced.

During the second exercise (Table 2), 13 subjects with poor execution (spine deviation exceeding the limit of 30°) were detected (in red) by both systems (OPT and IMU). Only one subject with a deviation close to the limit was considered poor by IMU and not by OPT. The 10 subjects with a high pelvic tilt (more than 65°, in blue) showed a reduced spine deviation (less than 21.5° with OPT), and nine of them were women. Seven of the eleven subjects with a leg rotation higher than 50° (in yellow) in the squat movement were inexperienced. The mean among subjects of the maximum trunk angular velocity was 232 deg/s for the swing kettlebell against 175 degree/s for the deadlift.

Since differences were found depending on gender and level of experience, a deeper analysis based on the results obtained from the optical motion system was conducted. Table 3 shows the mean values of the variables obtained for the three levels of experience. In both exercises, beginners (level A) presented a higher spine deviation, a lower pelvic tilt and a longer squat movement. However, there were only a statistically significant differences (*p* < 0.05) between subjects with less experience and those with more experience when it came to having a longer squat movement in both exercises, a higher spine deviation during the American kettlebell swing, and a lower pelvic tilt during the deadlift (Table 4).

Table 5 shows the mean values of the variables obtained for the two genders. In both exercises, women had better lifting technique, lower spine deviation, higher pelvic tilt and a shorter squat movement. All these hypotheses were statistically demonstrated (*p* < 0.05) by a two sample t-test, as shown in Table 4. It can be added that the mean initial pelvic tilt (taken as 0º during the motion) was 17.9° for women and 15.6° for men.

Finally, it must be said that measurements from inertial sensors showed good accuracy with RMSE of the maximum angle measurements of 2.71° and 3.01° during the American kettlebell swing and the deadlift, respectively, for the spine deviation (using two sensors), and 2.44° and 1.85° for the pelvic tilt (using one sensor). The RMSE for the spine deviation along the motion was 2.92° during the American kettlebell swing and 2.53° during the deadlift. Mean measurements were higher with inertial sensors, but there was only a statistically significant difference (*p* < 0.005) for the spine deviation during the deadlifts.

## 4. Discussion and Limitations of the Study

This work is a preliminary study aimed at developing a low-cost, wearable system composed of two IMUs for preventing lower back injuries and educating athletes about the hip hinge with neutral spine during lifting exercises. The authors analyzed the motion of 39 voluntary subjects (from several sport centers and with different ages, genders, and levels of experience) during the performance of two lifting exercises (the American kettlebell swing and the deadlift) to assess the feasibility and usefulness of the tool. The accuracy of the measurements was evaluated by comparing the obtained results with those from an optical motion capture system. In addition, the recorded motions were analyzed to identify the most relevant parameters of a good and safe lifting execution.

The results showed that more than 50% of common athletes have a poor spine posture during lifting exercises (during at least one of the two exercises captured), exceeding 30° of deviation from the neutral configuration, and presenting a high risk of lower back injury. Disc herniations will likely occur when doing a high number of repetitive cycles of flexion under relatively low loads around the limits of the neutral zone [2], and increasing the load will increase the likelihood of herniation development [32]. The mean spine deviation during deadlifts was 25.76° for the 39 subjects. Aasa et al. reported, in a similar study, an average lumbar flexion of 28° for 14 experienced powerlifters [21]. This study revealed that the level of experience is not decisive for safe lifting execution, because poor spine posture was detected for all categories. Some athletes and coaches were surprised by the results because their visual perception was different. In fact, visual observation does not take into account the differences in spine shapes [25] (clothing further complicates this assessment), and the speed of execution of the exercises makes the assessment difficult. Although the excessive squat error appears to be attributed to inexperience, pelvic tilt range of motion appears to be more gender related.

This work demonstrated that women have better lifting technique, with lower spine deviation, higher pelvic tilt and a lower squat movement. A recent study also found that males generally demonstrated less hip range of motion than females [33], which can be explained by the higher acetabular version in women [34]. For the 39 subjects analyzed, the mean initial pelvic tilt was 17.9° for women against 15.6° for men, which supports previous findings where a difference of 3° in the anteversion between genders was estimated [35].

Likewise, the results showed that people with a high pelvic tilt range of motion are less likely to have poor posture during lifting exercises. This fact supports the common limiting factor of the lumbopelvic dissociation: flexing the hips without moving the lower back constitutes a challenge for many people [24]. The tested wearable system composed of two IMUs could be very helpful to offer an objective feedback of pelvic tilt and body alignment to athletes so as to overcome this problem, allowing them to lift weights without suffering lower back injuries.

The accuracy of the IMUs, obtained by comparing the results with those from the optical motion capture system was satisfactory, with a mean RMSE along the motion lower than 3°, both for the fast movement of the kettlebell swing and for the large-range hip movement of the deadlift. Punchihewa et al. reported a RMSE of 5° by doing a similar comparison during baseball hitting [13]. It must be noted that the optical motion capture system presented many disadvantages during the study: a lot of elements to bring and set up (cameras, tripods, cables, etc.), a large space required to obtain a limited capture area, and manual post-processing needed due to occlusions of markers and other reflective objects misidentified as markers (sports clothes often have reflective parts).

Therefore, it can be said that the low-cost wearable system could accurately assess pelvic tilt and spine deviation when lifting weight, and could offer objective feedback to gym members and coaches to prevent injuries. However, because the inertial sensors tend to overestimate the measurements, it is recommended that the maximum accepted lumbar flexion is increased to 33° in order to avoid misjudging correct postures. Lastly, the other limitation of this study was sensor placement, which was carried out by means of a belt (sensor at vertebra L5) and a harness (sensor at vertebra T8). Authors are aware that this detail can lead to some errors, but the objective was to provide a system usable by gyms.

## 5. Conclusions and Future Works

The tested wearable system composed by the two IMUs demonstrated objective feedback on pelvic tilt and body alignment to gym members and coaches, thus helping to improve the hip hinge with lumbopelvic dissociation and to lift weights without suffering lower back injuries. This study showed that 18 of the 39 captured subjects were lifting weights with a poor spine posture (spine deviation >30° during, at least, one of the two exercises). They were warned of the danger of improper performance. Most of them thought they were doing it right, and some of them had been doing it wrong for years. This first contact with users and coaches enabled us to make them aware of the risk of lower back injuries when lifting weights, and the volunteers are now aware of their incorrect posture and will try to correct it with their trainer. Future studies will consist of developing an interface between the user and the sensors in order to provide live feedback and observing gym members’ acceptance and improvements. Furthermore, the tool could be used for ergonomics and occupational risk prevention as a lighter and more accurate tool than the lumbar motion monitor (LMM) [36] to prevent work-related musculoskeletal disorders.

## Figures and Tables

**Table 1:** Experimental measurements obtained for the American kettlebell swing (lower back flexion >30° in red and <20° in green; pelvic tilt >60° in blue; leg rotation >50° in yellow).

		American Kettlebell Swing				
		Max. Lower Back Flexion (°)		Max. Pelvic Tilt (°)		Max. Leg Rotation (°)
	Experience	OPT	IMU	OPT	IMU	OPT
**Women**	**A**	20.48	17.21	54.01	57.66	34.49
	**A**	31.47	36.12	57.03	56.65	82.36
	**A**	24.33	27.29	36.80	34.36	76.03
	**B**	32.19	30.48	50.99	44.34	26.90
	**B**	22.13	27.64	46.12	46.08	22.18
	**B**	26.00	31.11	52.66	52.84	33.01
	**B**	33.37	36.21	26.27	27.44	39.70
	**B**	20.81	26.25	54.34	54.02	16.17
	**B**	13.13	13.84	27.51	28.14	39.72
	**B**	19.26	19.08	58.08	61.74	34.82
	**B**	36.88	39.81	46.59	47.94	35.33
	**B**	3.25	5.68	54.89	56.68	27.80
	**B**	19.78	23.91	63.39	66.07	21.76
	**C**	28.85	32.64	47.73	47.15	26.13
	**C**	33.91	33.51	50.66	55.39	41.14
	**C**	28.36	28.47	44.26	46.02	47.34
	**C**	21.98	24.65	74.45	76.13	34.38
	**C**	22.96	25.97	38.16	41.16	29.37
**Men**	**A**	60.90	60.59	46.20	47.51	55.24
	**A**	20.70	19.14	58.88	60.81	73.50
	**A**	27.95	29.15	52.63	53.49	41.98
	**A**	45.39	49.12	37.28	37.65	46.06
	**A**	21.74	26.78	66.53	64.45	40.75
	**A**	32.29	33.84	18.93	20.70	51.17
	**B**	25.07	26.93	60.45	61.07	38.07
	**B**	17.41	20.04	40.69	43.81	38.58
	**B**	24.34	29.68	56.35	55.01	16.47
	**C**	47.78	42.99	58.44	59.60	40.24
	**C**	26.69	31.13	54.58	54.56	87.64
	**C**	21.38	17.37	57.39	51.84	42.64
	**C**	16.91	19.46	43.98	45.12	36.31
	**C**	16.99	19.37	57.41	57.61	22.30
	**C**	22.80	26.55	51.18	52.13	33.49
	**C**	25.82	29.35	33.42	36.85	40.63
	**C**	23.57	27.80	39.71	39.40	46.56
	**C**	39.24	40.65	18.14	18.32	34.23
	**C**	36.04	35.76	35.87	40.81	47.60
	**C**	24.17	22.42	48.42	50.57	30.44
	**C**	27.20	32.07	39.55	41.20	29.59
	**Mean**	**26.76**	**28.72**	**47.69**	**48.52**	**40.05**
	RMSE	2.71		2.44		

**Table 2:** Experimental measurements obtained for the deadlift (lower back flexion >30° in red and <20° in green; pelvic tilt >60° in blue; leg rotation >50° in yellow).

		Deadlift				
		Max. Spine Deviation (°)		Max. Pelvic Tilt (°)		Max. Leg Rotation (°)
	Experience	OPT	IMU	OPT	IMU	OPT
**Women**	**A**	25.03	22.84	58.30	64.18	58.46
	**A**	33.79	33.92	47.87	51.21	99.78
	**A**	22.37	21.51	52.06	50.51	67.83
	**B**	21.49	21.32	66.65	64.91	35.88
	**B**	20.78	24.05	68.40	67.06	15.10
	**B**	33.43	36.16	32.91	32.72	58.11
	**B**	24.06	26.47	37.02	38.07	47.34
	**B**	13.38	14.70	70.23	70.30	26.75
	**B**	33.38	36.04	33.65	32.92	36.56
	**B**	12.96	14.10	53.82	55.77	43.05
	**B**	21.93	23.50	73.82	74.94	32.30
	**B**	14.86	15.86	67.85	69.40	33.08
	**B**	21.58	26.81	70.01	70.12	20.86
	**C**	23.22	26.72	60.68	59.91	20.21
	**C**	4.19	9.32	65.38	64.15	30.85
	**C**	30.33	32.72	39.41	39.90	41.41
	**C**	18.60	18.51	83.06	86.39	27.44
	**C**	20.79	23.07	84.35	84.98	23.80
**Men**	**A**	56.41	61.77	29.13	29.08	106.06
	**A**	18.29	18.72	67.34	67.77	35.89
	**A**	10.71	9.32	58.06	57.10	70.87
	**A**	33.83	36.57	57.05	56.43	50.78
	**A**	18.70	22.52	49.82	50.47	53.73
	**A**	27.28	28.21	51.82	52.97	41.08
	**B**	19.88	22.71	62.48	61.59	32.86
	**B**	34.71	41.54	50.77	49.27	33.53
	**B**	44.29	48.77	55.22	55.75	21.83
	**C**	34.51	39.05	62.18	61.54	41.36
	**C**	19.26	25.20	38.74	34.81	78.90
	**C**	15.50	17.36	61.48	64.12	26.97
	**C**	36.27	38.65	40.07	41.93	38.74
	**C**	15.83	19.76	55.26	54.67	28.71
	**C**	26.90	29.86	61.27	60.54	38.33
	**C**	19.85	22.20	57.05	58.27	30.23
	**C**	33.39	35.54	46.32	47.66	61.66
	**C**	52.39	52.80	27.58	28.12	35.65
	**C**	29.44	30.53	60.13	62.11	37.85
	**C**	27.25	26.50	41.48	45.42	67.70
	**C**	33.59	35.44	62.75	63.13	33.04
	**Mean**	**25.76**	**27.97**	**55.42**	**55.90**	**43.19**
	RMSE	3.01		1.85		

**Table 3:** Mean values for levels of experience (lower back flexion >30° in red and leg rotation >50° in yellow).

		Swing KB (OPT)			Deadlift (OPT)		
		A	B	C	A	B	C
**Mean Spine Deviation (º)**		31.69	22.59	27.33	27.38	24.36	25.96
**Mean Max. Pelvic Tilt (º)**		47.59	49.10	46.67	52.38	57.14	55.72
**Mean Max. Leg Rotation (º)**		55.73	30.04	39.41	64.94	33.64	38.99

**Table 4:** *p*-value for several hypotheses (*p* < 0.05 in red).

	*p*-Value					
	American Kettlebell Swing			Deadlift		
H0	Max. Spine Deviation	Max. Pelvic Tilt	Max. Leg Rotation	Max. Spine Deviation	Max. Pelvic Tilt	Max. Leg Rotation
**W=M**	0.000392	0.032860	0.000681	0.000002	0.000018	0.000676
**A=B**	0.000293	0.351078	0.000000	0.079142	0.011574	0.000000
**B=C**	0.001229	0.069403	0.000028	0.207485	0.320128	0.002534
**A=C**	0.014508	0.565281	0.000011	0.356411	0.048909	0.000001
**OPT=IMU**	0.01206	0.34031	/	0.005563	0.574423	/

**Table 5:** Mean values for genders.

		Swing KB (OPT)		Deadlift (OPT)	
		W	M	W	M
**Mean Spine Deviation (º)**		24.40	28.78	22.01	28.97
**Mean Max. Pelvic Tilt (º)**		49.11	46.48	59.19	52.19
**Mean Max. Leg Rotation (º)**		37.15	42.55	39.93	45.99

## Data Availability

The data presented in this study are available on request from the corresponding author. The data are not publicly available due to privacy restrictions.
